# Limit cycles in models of circular gene networks regulated by negative feedback loops

**DOI:** 10.1186/s12859-020-03598-z

**Published:** 2020-09-14

**Authors:** Vitaly A. Likhoshvai, Vladimir P. Golubyatnikov, Tamara M. Khlebodarova

**Affiliations:** 1 Department of Systems Biology, Institute of Cytology and Genetics, Siberian Branch RAS, Novosibirsk, Russia; 2 Laboratory of Inverse Problems of Mathematical Physics, Sobolev Institute of Mathematics Siberian Branch RAS, Novosibirsk, Russia; 3 Novosibirsk State University, Novosibirsk, Russia

**Keywords:** Mathematical modeling, Circular gene networks, Delay argument equations, Feedback loops regulation, Autorepressor, Cycles, Phase portraits, Inverse problems

## Abstract

**Background:**

The regulatory feedback loops that present in structural and functional organization of molecular-genetic systems and the phenomenon of the regulatory signal delay, a time period between the moment of signal reception and its implementation, provide natural conditions for complicated dynamic regimes in these systems. The delay phenomenon at the intracellular level is a consequence of the matrix principle of data transmission, implemented through the rather complex processes of transcription and translation.However, the rules of the influence of system structure on system dynamics are not clearly understood. Knowledge of these rules is particularly important for construction of synthetic gene networks with predetermined properties.

**Results:**

We study dynamical properties of models of simplest circular gene networks regulated by negative feedback mechanisms. We have shown existence and stability of oscillating trajectories (cycles) in these models. Two algorithms of construction and localization of these cycles have been proposed. For one of these models, we have solved an inverse problem of parameters identification.

**Conclusions:**

The modeling results demonstrate that non-stationary dynamics in the models of circular gene networks with negative feedback loops is achieved by a high degree of non-linearity of the mechanism of the autorepressor influence on its own expression, by the presence of regulatory signal delay, the value of which must exceed a certain critical value, and transcription/translation should be initiated from a sufficiently strong promoter/Shine-Dalgarno site. We believe that the identified patterns are key elements of the oscillating construction design.

## Introduction

Molecular-genetic systems or gene networks are molecular machines which decode genetic programs and bring them up to all the levels of living systems organization, from the molecular level (metabolic networks, signaling pathways, splicing, transport etc.) to subcellular, cellular, tissue, and organismic one. During the evolution, these systems have obtained ability to adequate reaction on the external signals and on changing of the environmental conditions. Their structure is based on regulatory circuits, i.e., on sub-networks composed by genes, which encode transcriptional factors and some types of enzymes (for example kinase), and their different combinations configure gene networks regulated by positive, negative, or variable feedback mechanisms.

From the evolutionary viewpoint, configuration of loops with negative feedback loops regulation is more simple task for a cell, than that of loops with positive feedback loops, because it can be accomplished by a simple blockage of the transcription initiation site by the regulatory protein without formation of complex structures that interact with the RNA polymerase.

**Fig. 1:**
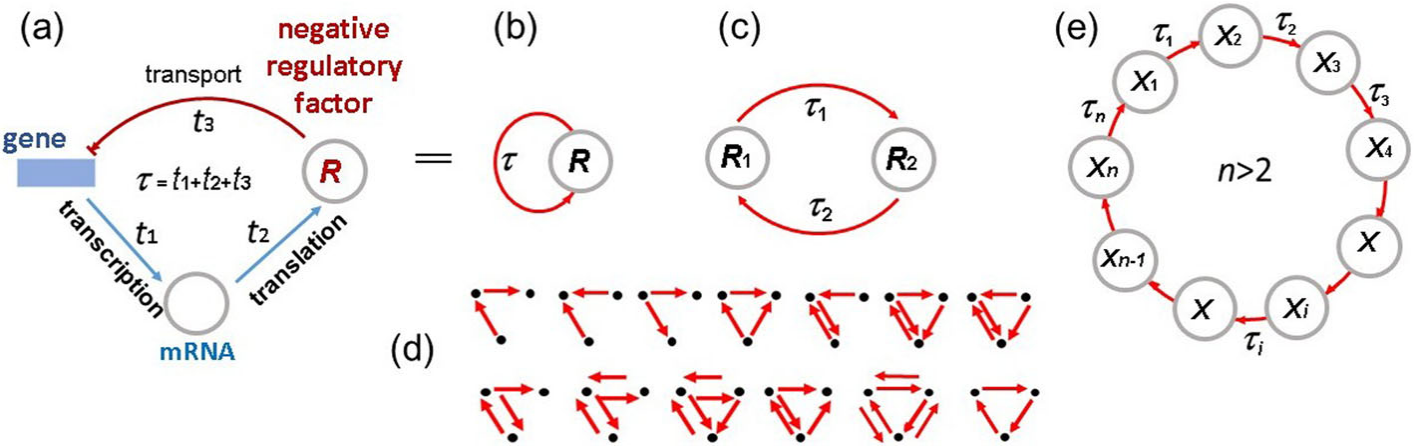
Simplest autorepressilators: **a** negatively autoregulated one-gene network, **b** its graphical scheme, **c** graph of two-genes network, **d** variety of sub-networks in a trigenic network, **e** graph of n-elements gene network

This type of regulation is widespread in nature, and its simplest example is a subsystem containing just one gene which controls its own expression by negative feedback mechanism. This is the minimal regulatory circuit, it acts on itself (Fig. 1a,b), and there exists a great amount of such regulatory subsystems. For the prokaryotes, in particular for *E.coli*, this regulation mechanism is typical for global regulators, such as FNR (fumarate nitrate reduction regulator) and CRP (cAMP receptor protein) [1–3] for stress response regulators LexA (locus for X-ray sensitivity A) and MarR (multiple antibiotic resistance regulator) [4, 5], and for transcriptional factors H-NS (histone-like nucleoid structuring protein), IHF (integration host factor), Fis (factor for inversion stimulation), Lrp (leucine-responsive regulatory protein) etc [6–9]. A lot of similar examples is known for the eukaryotes as well. This type of regulation is characteristic for RB (retinoblastoma) gene [10], hes family basic helix-loop-helix factor (bHLH) transcription factors, which are important for coordinated somite segmentation [11, 12], DNA binding homeobox transcription factor Nanog, which is involved in embryonic stem cell proliferation, renewal, and pluripotency [13, 14], and many other genes [15–18], etc.

The next stage of regulatory circuits’ complexity is represented by the two-gene systems, where each gene encodes expression regulator of another gene (Fig. 1c). Here, the examples of negative regulation circuit are pairs of regulatory factors Sox3-Snail and DDX21-Snail. They are repressors of each other expression and control formation of ecto- and mesoderm at early stage of embryo development, [19]. For the *Bacillus subtilis*, such properties have products of the genes *ComK* (competence transcription factor) and *kre* (ComK repressor) which participate in differentiation of cellular phenotypes [20]. There are similar systems regulated by RNA molecules, for example non-coding RNA MicF and global regulator of *E.coli* metabolism Lrp act negatively on expression of each other [21, 22].

The following class of complexity is composed by trigenic regulatory circuits, where each gene encodes transcriptional factor, or another protein, and regulation is the circular one. An example of such a system is negative regulatory circuit AMPK-mTOR-NRF2 which acts in regulation processes of autophagy [23].

Studies of dynamical characteristics of circular gene networks, in particular networks regulated by negative feedback loops, are motivated not only by their prevalence in molecular genetic systems, which is important per se, but also by their role in dynamics of different biological processes. Even the simplest regulatory circuits incorporated into complicated gene networks can determine dynamics of the process in the large, as it was shown for the gene *HES*7, whose oscillating expression dynamics determines process of vertebrata somitogenesis and is related with its autorepression properties [24].

Now, we study only simplest circular regulatory circuits which can be recovered in natural gene networks. This is specified by the rapid increase of the amount of subsystems when the amount of genes grows. For example, if the number *n* of gene elements in a circular gene network equals 3, then the number *s*(3) of its sub-networks equals 13 (Fig. 1d). If *n*=4, then *s*(4) equals 201, if *n*=5, then *s*(5)=9390; similarly *s*(6)=1531336,*s*(7)=880492496,*s*(8)=1792477159408 etc. So, the mathematical analysis of these subsystems seems to be technically unfeasible.

We do not consider here cyclic networks regulated by mixed feedback mechanisms, which are positive and negative at the same time. The models of these gene networks possess not only a high oscillatory potential [25–27] but also the possibility of complex, chaotic dynamics formation [28–31], including the hyperchaotic one [32–34]. Here we consider mathematical models of natural circular gene networks. Our studies are based both, on numerical simulations of these networks and on analytic mathematical methods. We describe limit cycles in simplest circular gene networks models and elaborate algorithms of construction of these cycles.

## Results

### Analysis of limit cycles in a model of the simplest autorepressilator

The model of simplest circular regulatory circuit which acts by the negative feedback loop is represented by an equation

1 $$ \frac{d x(t)}{dt} = f(x(t-\tau)) - x(t); \quad f(x) = \frac{\alpha}{1+x^{h}}.  $$

Description and interpretation of this equation and that of positive parameters *α* and *h* are given below in the “Methods” section.

It was shown in [35] that the Eq. (1) has exactly one positive equilibrium point *x*_0_>0, where $x_{0}(1+x_{0}^{h})= \alpha $. If *τ*=0, then for all values of parameters *α* and *h* this equilibrium is stable, and all trajectories of the Eq. (1) tend to *x*_0_. However, if $w=h \frac {x_{0}^{h}}{1+x_{0}^{h}} >1, u^{2}=w^{2}- 1$, and $ \tau \geq \tau ^{*}=\frac {\pi - \arctan (u)}{u}$ then the equilibrium point becomes unstable and a limit cycle with period $T(\tau ^{*})=\frac {2 \pi }{u}$ appears there, see [35].

It follows from the numerical experiments that for *τ*>*τ*^∗^, the Eq. (1) has only one stable cycle, and its period *T*(*τ*) exceeds 2*τ*. So, we assume that this equation does not have other limit cycles with periods greater than 2*τ*, though we have not proved this analytically.

At the same time, this Eq. (1) can possess cycles whose periods are less than *τ*. Actually, for *τ*≥*τ*^∗^ and any natural *m*, there exists at least one limit cycle such that *τ*^∗^+*mT*(*τ*^∗^)≤*τ*. This limit cycle is constructed as follows: let *S*_*m*_(*τ*^′^)=*τ*^′^+*mT*(*τ*^′^), where *T*(*τ*^′^) is the period corresponding to the parameters *α*,*h*,*τ*^′^. Numerical analysis of these cycles shows that this function grows monotonically with *τ*^′^. When *τ*^′^ grows from *τ*^∗^ to *τ*, we have *S*_*m*_(*τ*^∗^)≤*τ* and *S*_*m*_(*τ*)>*τ*. So, we see that if for some natural *m* the inequality *τ*^∗^+*mT*(*τ*^∗^)≤*τ* holds, then the Eq. (1) with the parameters *α*,*h*,*τ*_*m*_ has a limit cycle corresponding to the parameters *α*,*h*,*τ*.

### Limit cycles of gene networks models for *n*>1

Natural generalization of the model (1) to the case of circular gene network with *n* genetic elements (Fig. 1e) has the form:

2 $$ \frac{d x_{1}}{dt} = \frac{\alpha_{1}}{1+x_{n}^{h_{n}} (t - \tau_{n})} - \beta_{1} x_{1}, \, \frac{d x_{i}}{dt} = \frac{\alpha_{i}}{1+x_{i-1}^{h_{i-1}} (t - \tau_{i})} - \beta_{i} x_{i}, \,\,\, i=2,3,\ldots n.  $$

As in (1), the functions $ f_{i}(x) = \frac {\alpha _{i}}{1+x^{h_{i}}} $ describe negative feedback mechanisms in the gene network, *x*_1_=*x*_1_(*t*),*x*_*i*_=*x*_*i*_(*t*) are concentrations as above. For *n*=2, the system of equations of the type (2) is the simplest model of artificial molecular trigger [36, 37], for *n*=3, this is a model of Elowitz-Leibler repressilator [38], see also [39, 40] for more biological interpretations. Detailed mathematical analysis of delayed arguments phenomena in similar models is given in [41]. For its piece-wise version, the sufficient and necessary conditions of existence of a limit cycle were obtained in [42]. For similar 5-dimensional system, sufficient condition of existence of a cycle was obtained in [43], where some results on its stability were presented as well.

In our numerical experiments, we have seen that if the system (2) with the parameters *α*_*i*_, *β*_*i*_, *h*_*i*_, *τ*_*i*_ has a limit cycle *C*_1_ with coordinate functions (*x*_1_(*t*),*x*_2_(*t*),…,*x*_*n*_(*t*)), then the same system with the parameters $ \alpha _{i}, \beta _{i}, h_{i}, \bar {\tau }_{i} $ such that $ {\sum \nolimits }_{i=1}^{i=n} \tau _{i} = {\sum \nolimits }_{i=1}^{i=n} \bar {\tau }_{i} $, has a limit cycle *C*_2_(*y*_1_(*t*),*y*_2_(*t*),…*y*_*n*_(*t*)) whose coordinate functions *y*_*i*_(*t*) coincide with *x*_*i*_(*t*) for all *i* up to some shift of *t*. So, one can assume here that $ {\sum \nolimits }_{i=1}^{i=n} \tau _{i} < T $, where *T* is the period of the cycles *C*_1_,*C*_2_, and that *τ*_1_=*τ*_2_=…=*τ*_*n*_.

### The case of infinite value of the parameter *h*

When *h*_*i*_→*∞* for all *i*, then the system (2) reduces to the following one:

3 $$ \frac{d x_{1} (t)}{dt} = L_{1} (x_{n} (t-\tau_{1}))- \beta_{1} x_{1} (t); \quad \frac{d x_{i} (t)}{dt} = L_{i} (x_{i-1} (t-\tau_{i}))- \beta_{i} x_{i} (t);  $$

where *L*_*i*_ are the step functions: *L*_*i*_(*x*)=*β*_*i*_*α*_*i*_ for 0≤*x*≤1; *L*_*i*_(*x*)=0 for *x*>1. Similar systems were studied in [41–43] in analysis of circular gene networks, *i*=2,3,…,*n*.

**Fig. 2:**
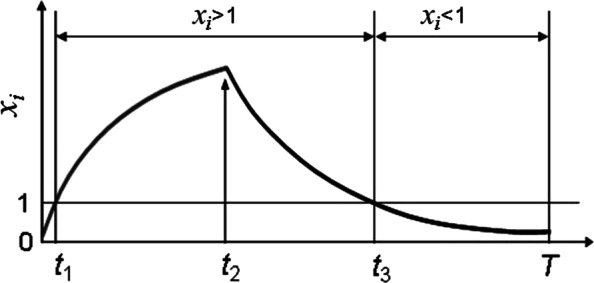
Standard form of the graphs of the solutions *x*_*i*_(*t*) of the system (3). The horizontal axis is *t*, the vertical lines *t*_1_,*t*_3_ correspond to *x*_*i*_(*t*)=1

The graphs of periodic solutions *x*_*j*_(*t*) of the system (3) have a standard form, see the Figure 2.

We consider now the symmetric simplified version of the system (3) in the case *α*_1_=*α*_2_=…=*α*_*n*_=*α*,*β*_1_=*β*_2_=…=*β*_*n*_=1, in order to describe all periodic trajectories of the system (3) which are symmetric with respect to the cyclic permutations of the variables *x*_1_→*x*_2_→…→*x*_*n*_→*x*_1_. In this case this system is reduced to just one equation with constant delay *τ*:

4 $$ \frac{dx}{dt} = \alpha - x(t) \,\,\,\, \text{for} \,\,\,\, x(t-\tau) < 1; \quad \frac{dx}{dt} = - x(t) \,\,\,\, \text{for} \, \,\,\, x(t-\tau) > 1;  $$

Its solution has the form:

5 $$ \begin{aligned} & x(t) = (x(t_{i}) - \alpha) e^{-(t_{i} - t)} + \alpha \,\,\,\, \text{if} \,\,\,\, x(t_{i}) - t = 1 \,\,\,\, \text{and} \,\,\,\, x(t-\tau) < 1; \\ & x(t) = x(t_{i}) e^{-(t_{i} - t)} \,\,\,\, \text{if} \,\,\,\, x(t_{i} - t) = 1 \,\,\,\, \text{and} \,\,\,\, x(t-\tau) > 1. \end{aligned}  $$

We assume here that 0<*t*_1_<*t*_2_<…<*t*_*k*_<*T* is partition of the period [0,*T*] to intervals (*t*_*i*_,*t*_*i*+1_) where either *x*(*t*−*τ*)<1, or *x*(*t*−*τ*)>1.

If $ \tau = \ln \frac {\alpha + \sqrt {\alpha ^{2} + 4(e^{T} - 1)(\alpha -1)}} {2 \alpha }$ then the Eq. (4) has a limit cycle *C*_1_ with the period *T*, and such *τ* is unique on the interval (0,*T*/2). In this case characteristics of the graph on the Fig. 2 have the form

6 $$ \begin{aligned} & t_{1}= \ln \frac{\alpha - e^{-\tau}}{\alpha - 1}, \quad t_{2}= \ln \frac{\alpha e^{\tau} - 1}{\alpha - 1}, \quad t_{3}=T-\tau, \,\, \\ & x_{0} =x(0)=x(T)=e^{-\tau}, \quad x(t_{2})= \frac{e^{\tau} \alpha - \alpha+1}{e^{\tau}}. \end{aligned}  $$

Numerical analysis of this cycle shows that it is stable.

Now, fix *τ*>0, and *α*>1, then the Eq. (4) has a limit cycle *C*_2_ with the period *T*(*τ*) such that

7 $$ T(\tau)= \ln \frac{(\alpha e^{\tau} - \alpha +1)(\alpha e^{\tau} -1)} {\alpha-1}.  $$

This is unique limit cycle such that *T*(*τ*)>2*τ*. Surely, if this condition *T*(*τ*)>2*τ* is satisfied, then the cycles *C*_1_ and *C*_2_ coincide.

#### **Lemma 1**

If *T*(*τ*)<2*τ*, then the Eq. (*4*) has a countable set of limit cycles.

#### *Proof*

Let *k*≥1 be a natural number. Consider *τ*_*k*_<*τ* such that *T*(*τ*_*k*_) calculated by the formulae (6), (7) satisfies the condition *τ*_*k*_+*kT*(*τ*_*k*_)=*τ*. Then the equation

8 $$ e^{T(\tau_{k})} = \frac{\left(\alpha e^{\tau-kT(\tau_{k})} - \alpha +1\right) \left(\alpha e^{\tau - kT(\tau_{k})} -1\right)}{\alpha-1}; \quad \tau_{k} = \tau - kT(\tau_{k}) > 0  $$

has a unique positive solution *T*(*τ*_*k*_). □

So, we have constructed a countable set of limit cycles {*C*_*k*_} such that, given *k*, the relations (8) determine the cycle *C*_*k*_, and the lemma is proved.

Now, we give a description of a symmetric limit cycle of *n*-dimensional system (4) with the paraemter *α*. Let *T* be the period of this cycle, and (*x*_1_(*t*),*x*_2_(*t*),…,*x*_*n*_(*t*) be its coordinate *T*-periodic functions. Since the cycle is symmetric, we have

$$x_{i} (t) = x_{i+1} \left(t+ \frac{p}{n}T\right), \qquad x_{n} (t) = x_{1} \left(t+ \frac{p}{n}T\right). $$

Here, *i*=1,2,…,*n*−1, and positive natural *p* does not exceed (*n*−1). Thus, each positive solution of the equation

$$e^{T(\tau_{n,p,k})} = \frac{\left(\alpha e^{\tau + (p/n - k) T(\tau_{n,p,k})} -\alpha +1\right) \left(\alpha e^{\tau + (p/n - k) T(\tau_{n,p,k})} -1 \right)}{\alpha - 1}, $$

9 $$ \tau_{n,p,k} = \tau + \left(\frac{p}{n} -k \right) T(\tau_{n,p,k}) > 0, \,\,\, p= 1,2,\ldots n-1, \,\, k=0,1, \ldots \left[ \frac{\tau}{T(\tau_{n,p,k})} + \frac{p}{n} \right]  $$

corresponds to a limit cycle of the system (4), and given $ \tau > 0, T(\tau _{n,p_{1},k_{1}}) = T(\tau _{n,p_{2},k_{2}}) $ if and only if *k*_1_=*k*_2_, and *p*_1_=*p*_2_.

### The case absence of delay arguments

In the particular case of the system (3) with *τ*_1_=*τ*_2_=…=*τ*_*n*_=0, it was shown in [44], that for odd values of *n* such a system has a limit cycle if and only if *α*_*i*_>1 for all *i*=1,2,…,*n*. For *n*=3 this cycle is unique and stable [45].

If *n*=5,*β*_1_=*β*_2_=…=*β*_5_=1, all the functions *L*_*i*_ coincide, and $2 \alpha >5+\sqrt {5}$, then the system (4) has at least two limit cycles, see [46].

For even values of *n*, no stable cycles have been discovered in our studies, both numerical and analytical.

Consider an inverse problem of identifications of parameters of 3-dimensional dynamical system

10 $$ \frac{dx(t)}{dt}=L_{1} (z)-x(t); \,\, \frac{dy(t)}{dt}=L_{2} (x)-y(t); \,\, \frac{dz(t)}{dt}=L_{3} (y)-z(t),  $$

where *L*_*i*_ are as in (3), and *α*_*i*_>1,*i*=1,2,3.

Piecewise linear systems of the type (10) in different dimensions were studied in gene networks modeling earlier, see for example [47–49]. As it was shown in [50], the system (10) has a unique limit cycle *C* with the periodic coordinate functions *x*(*t*),*y*(*t*),*z*(*t*); let *T* be its period, and [0,*T*] be a segment on the *t*-axis subdivided by the points 0<*t*_1_<*t*_5_<*t*_2_<*t*_3_<*t*_4_<*T* so that the graph of the function *y*(*t*) is depicted on the Fig. 2. Unusual position of *t*_5_ is chosen specially, in order to refer to this Figure. So, *y*_0_=*y*(0)=*y*(*T*) is the minimal value of *y*(*t*),*y*_2_=*y*(*t*_2_) is its maximal value, and *y*(*t*_1_)=*y*(*t*_3_)=1. Respectively, *z*_1_=*z*(*t*_1_) is the maximal value of *z*(*t*),*z*_3_=*z*(*t*_3_) is its minimal value, *x*_5_=*x*(*t*_5_) is the minimal value of *x*(*t*), and *x*_4_=*x*(*t*_4_) is its maximal value. At the same time, *z*(*t*_4_)=*z*(*t*_5_)=*x*(0)=*x*(*t*_2_)=1. The shapes of the graphs of the functions *x*(*t*),*z*(*t*) are similar to that of *y*(*t*), though their maxima and minima are different as well, as the lengths of the segments in the partition of [0,*T*]. So, it follows from the formulae (5) that

11 $$ \begin{aligned} & z_{1} \cdot e^{-(t_{5}-t_{1})} =1; \,\,\, z_{1} \cdot e^{-(t_{3}-t_{1})} = z_{3}; \,\,\, (z_{3} - \alpha_{3})\cdot e^{-(t_{4}-t_{3})} + \alpha_{3} = 1; \\ & y_{2} \cdot e^{-(t_{3}-t_{2})} = 1; \,\,\,\, y_{2} \cdot e^{-(T-t_{2}) }=y_{0}; \,\,\,\, (y_{0} - \alpha_{2}) \cdot e^{-t_{1}} + \alpha_{2} = 1; \\ & x_{4} \cdot e^{-(T-t_{4}) }= 1; \,\, x_{4} \cdot e^{-(T-t_{4})- t_{5}}= x_{5}; \,\, (x_{5} - \alpha_{1}) \cdot e^{-(t_{2}-t_{5}) }+\alpha_{1} = 1, \end{aligned}  $$

and these relations imply the following proposition:

#### **Proposition 1**

If the moments *t*_*j*_ of extrema of the coordinate functions *x*(*t*),*y*(*t*),*z*(*t*) are known from observations of the limit cycle *C*, then these maximal and minimal values, and the parameters *α*_1_,*α*_2_,*α*_3_ can be expressed explicitly in terms of *t*_*j*_ from the Eq. (11).

For example,

$$z_{1} = e^{(t_{5}-t_{1}) }>1; \,\, z_{3} =e^{(t_{5}-t_{3})} <1; \,\, \alpha_{3} \left(1-e^{-(t_{4}-t_{3})}\right)= 1-e^{-(t_{4}-t_{5})} ; \quad \text{etc.} $$

Analogous inverse problems of identifications of parameters of higher-dimensional dynamical systems of the type (4) can be formulated and solved in a similar way, see for example, [51, 52].

### The case of finite values of *h*

#### **Lemma 2**

If the symmetric system

12 $$ \frac{d x_{1}}{dt} = \frac{\alpha}{1+x_{n}^{h} (t - \tau)} - x_{1}, \,\, \ldots \, \frac{d x_{i}}{dt} = \frac{\alpha}{1+x_{i-1}^{h} (t - \tau)} - x_{i}, \,\, i=2,3,\ldots n ;  $$

has a symmetric *T*-periodic trajectory with coordinate functions (*x*_1_(*t*),…,*x*_*n*_(*t*)), then

13 $$ x_{1} \left(t-\left(\tau+ \frac{p}{n}T \right) \right) = x_{n} (t), \ldots, x_{i} \left(t-\left(\tau+ \frac{p}{n}T \right) \right) = x_{i-1} (t), \quad i = 2,3,\ldots, n,  $$

where *p* is an integer such that 0≤*p*<*n*, and for each *j*,*j*=1,2,…,*n*, the function *x*_*j*_(*t*) is a *T*-periodic solution of the Eq. (*1*) with the parameters $ \alpha, h, \bar {\tau }=\tau + \frac {p}{n}T$. The converse is also true.

Thus, each symmetric limit cycle of the system (12) can be constructed from the periodic solution of (1) with parameters $ \alpha, h, \bar {\tau }=\tau + \frac {p}{n}T$, and we can formulate the following algorithm of construction of these symmetric limit cycles:

**Algorithm 1.**

It follows from Lemma 2 that in order to construct symmetric limit cycles of the system (12), one has to find *T*-periodic solutions of the equation

14 $$ \frac{d x(t)}{dt} = f(x(t- (\tau + \frac{p}{n}T)) - x(t); \quad f(x) = \frac{\alpha}{1+x^{h}},  $$

where *p*=1,2,…,(*n*−1). Then for any limit cycle *x*(*t*) of the Eq. (14), the system (12) has a limit cycle (*x*_1_(*t*),*x*_2_(*t*),…,*x*_*n*_(*t*)) such that

$$x_{i} (t) = x\left(t - i \left(\tau + \frac{p}{n}T \right) \right). $$

**Fig. 3:**
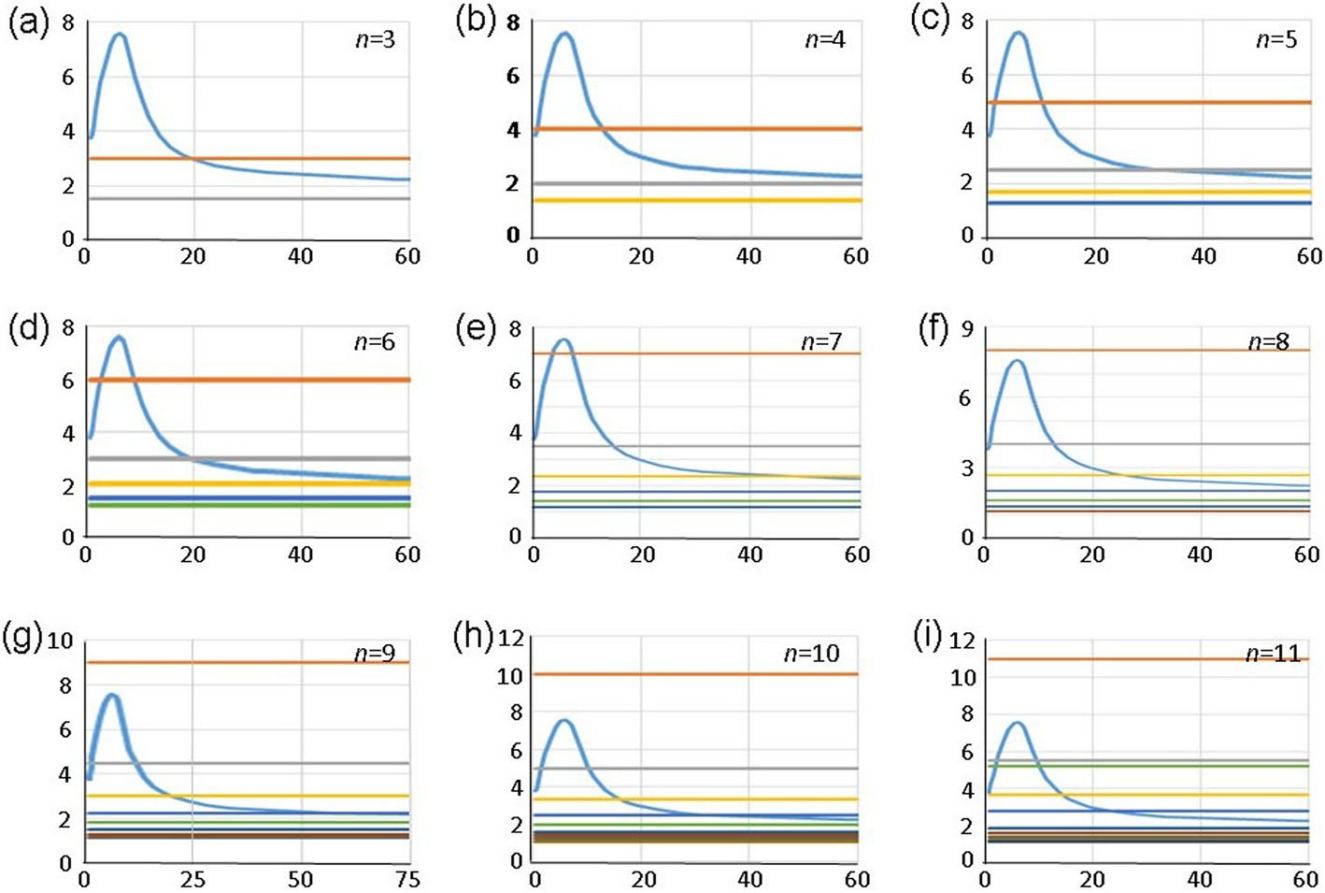
Geometric interpretation of the Algorithm 1 for *τ*=0. The blue curve is the graph of the function $ T\left (\frac {p}{n} \tau _{1} \right)$. The horisontal axis is *t*, the vertical one shows the values of $ T\left (\frac {p}{n} \tau _{1} \right)$ and $\frac {n}{p} $. On each graph *p*=1,2,…,*n*−1. **a** *n*=3; **b** *n*=4; **c** *n*=5; **d** *n*=6; **e** *n*=7; **f** *n*=8; **g** *n*=9; **h** *n*=10; **i** *n*=11

For autonomous sytems, when *τ*=0, this algorithm has the following illustrative presentation. Consider the graph of the function $y(\tau) = \frac {T(\tau)}{\tau }$, and fix any integer *p*, such that 0<*p*<*n*. Then for *T*(*τ*)>2*τ*, the limit cycle does exist, if the line $ y = \frac {n}{p}$ intersects the graph of *y*(*τ*), see the Fig. 3.

So, if $\frac {n}{p} \leq 2$, or $ \frac {n}{p} > \max _{\tau > \tau ^{*}} \frac {T(\tau)}{\tau }$ then the system (12) does not have limit cycles whose coordinate funcitions satisfy the relations (13).

If $\, \frac {n}{p} = \max _{\tau > \tau ^{*}} \frac {T(\tau)}{\tau }$, or $ 2 < \frac {n}{p} < \frac {T(\tau ^{*})}{\tau ^{*}} $, then there exists only one such cycle.

If $\, \frac {T(\tau ^{*})}{\tau ^{*}} \leq \frac {n}{p} < \max _{\tau > \tau ^{*}} \frac {T(\tau)}{\tau }$, then there exist two such cycles,

Consider as examples the limit cycles of *n*-dimensional systems, for *α*=1000,*h*=10,*n*=2,3,…,11. Each symmetric limit cycle of *n*-dimensional system (12) with the parameters *α*, *h*, and *τ*, can be obtained from *T*(*τ*)-periodic trajectories of the Eq. (1) with the parameters *α*, *h*, and *τ*.

For *n*=2 there are no symmetric limit cycles,

For *n*=3 there exists one symmetric limit cycle with the phase lag *T*/3, Fig. 3a.

For *n*=4 there exist two symmetric limit cycles with the phase lag *T*/4, Figure 2 b.

For *n*=5 there exist two symmetric limit cycles with the phase lag *T*/5 and one limit cycle with the phase lag 2*T*/5, Fig. 3c.

For *n*=6 there is one symmetric limit cycle with the phase lag *T*/3, and two limit cycles with the phase lag *T*/6, Fig. 3d.

For *n*=7 there is one limit cycle with the phase lag 3*T*/7, one limit cycle with the phase lag 2*T*/7, and two limit cycles with the phase lag *T*/7, Fig. 3e.

For *n*=8 there is one symmetric limit cycle with the phase lag 3*T*/8, and two limit cycles with the phase lag *T*/4, Fig. 3f.

For *n*=9 there is one symmetric limit cycle with the phase lag 4*T*/9, and two limit cycles with the phase lag 2*T*/9, Fig. 3g.

For *n*=10 there is one limit cycle with the phase lag 2*T*/5, one limit cycle with the phase lag 3*T*/10, and two limit cycles with the phase lag $ \frac {T}{5}$, Fig. 3h.

For *n*=11 there is one limit cycle with the phase lag 5*T*/11, one limit cycle with the phase lag 4*T*/11, two limit cycles with the phase lag 3*T*/11, and two limit cycles with the phase lag 2*T*/11, Fig. 3i.

Using the Algorithm 1, similar examples can be described for higher-dimensional versions of the system (12).

### On non-uniqueness of limit cycles in models of circular gene networks

Another algorithm of construction of symmetric limit cycles of the system (11) is based on factorization of the dimension *n*, see [53].

**Algorithm 2.**

Let *n*=*s*·*ℓ*, and *s*>1,*ℓ*>1 be such a factorization. If *s*-dimensional system (12) has a symmetric limit cycle *C*_*s*_(*x*_1_(*t*),…,*x*_*s*_(*t*)) such that *x*_*i*_(*t*)=*x*_*i*+1_(*t*−*T*_*s*_/*s*),*i*=1,…,*s*, then the *n*-dimensional system of the type (12) has a symmetric limit cycle *C*_*n*,*s*_(*x*_1_(*t*),…*x*_*n*_(*t*)) such that *x*_*j*_(*t*)=*x*_*j*+1_(*t*−*T*_*s*_/*s*). We assume here, as usual, that if *i*=*s*, then *i*+1=1, respectively, if *j*=*n*, then *j*+1=1.

#### **Proposition 2**

Let *C*_*n*_ be a symmetric limit cycle of *n*-dimensional system (*12*), and *x*_*j*_(*t*)=*x*_*j*+1_(*t*−*pT*_*n*_/*n*). This cycle *C*_*n*_ is irreducible if and only if *p* and *n* are coprime.

If *p* and *n* are not coprime, their greatest common divisor equals *m*, and *n*=*m*·*s*,*p*=*m*·*q*, then *C*_*n*_ is generated by the limit cycle *C*_*m*_ of *m*-dimensional system of the form (*12*) such that $x_{r} (t)=x_{r+1} \left (t-q \frac {T_{m}}{m} \right), r=1,2,\ldots,m$.

#### **Corollary 1**

Each symmetric limit cycle of the system (*3*) is either irreducible, or is generated by an irreducible cycle.

The limit cycle *C*_*n*,*s*_ is called reducible, and if the system (12) has a symmetric limit cycle which cannot be constructed by the Algorithm 2, we call it irreducible.

Numerical experiments show that limit cycle *C*_*n*_ is stable if and only if the dimension *n* is odd, *n*=2*m*+1, and $x_{j} (t)=x_{j+1} \left (t- m \frac {T_{n}}{n} \right)$. Here *j*=1,2,…,*n*, as above.

## Discussion

This paper is devoted basically to symmetric limit cycles in models of gene networks regulated by negative feedback loops only. Two algorithms of construction of these cycles are described. In this paper we have shown that

**Fig. 4:**
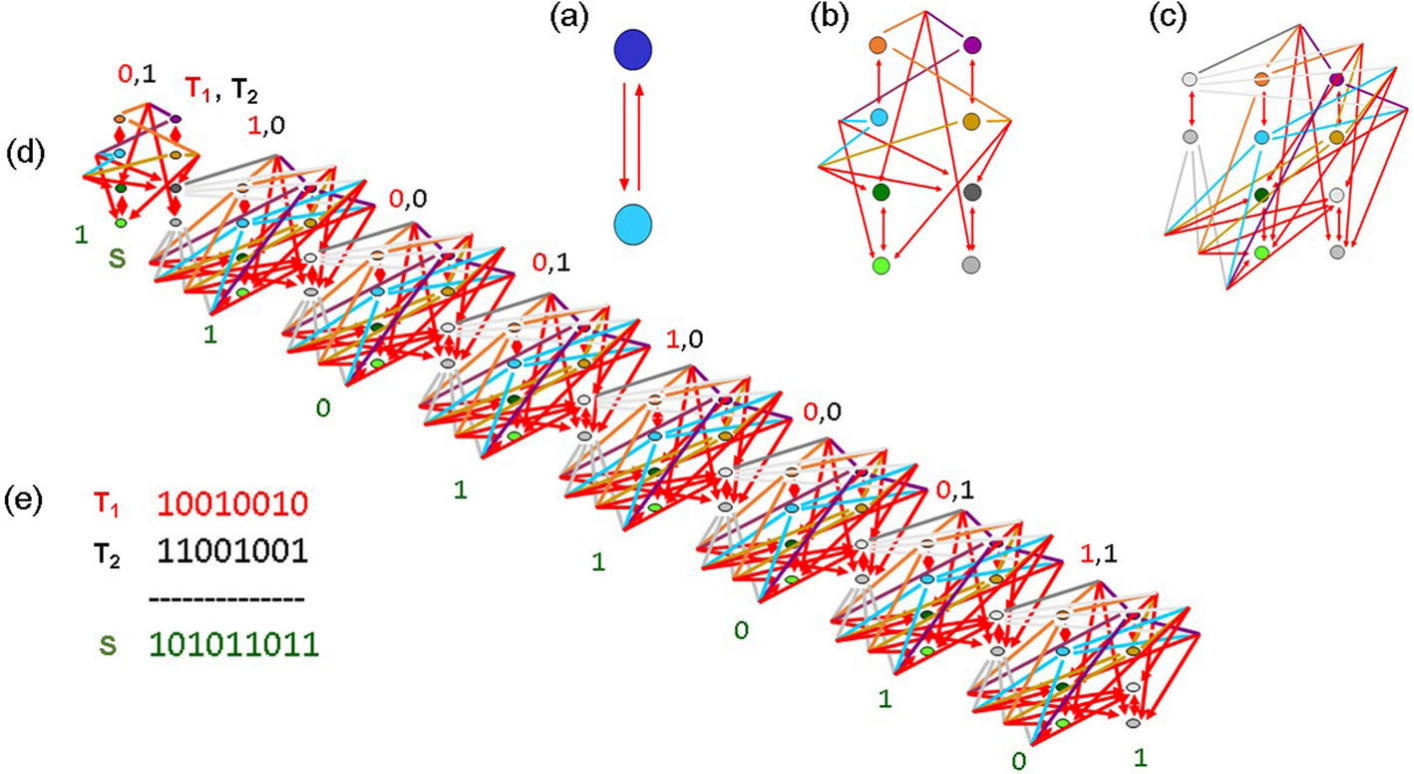
Hypothetic summating gene network; **a** memory cell; **b** Fragment 1 calculating the sum of the least lower order digits; **c** Fragment 2 calculating the sums of higher order digits; **d** summating gene network for *n*=8; **e** the states of the triggers in the summating process

1. (a) Natural gene networks, not necessary circular, have oscillating potential since usually they contain circular gene subnetworks.

(b) Initiation of transcription/translation processes in these networks is realized by sufficiently powerful promotor/SD-site: *α*>>1.

(c) Autorepressor’s action is nonlinear: *h*>1.

(d) For low-dimensional gene networks, the period between the initiation moment and the appearance of an active molecule in oscillations of their synthesis should exceed some critical value: *τ*>*τ*^∗^.

2. At the same time, for *n*=2, the systems (4) describe so called molecular triggers, and do not have limit cycles. Their analogues in the natural gene networks are genetic subsystems, that act as switches from one mode of operation to another under external conditions and/or regulatory signals [36, 37].

3. Such molecular triggers can realize in perspective memory elements in design and construction of artificial genetic programs. The main attractive property of these molecular triggers is their bistability, and absence of limit cycles makes these systems more stable. As an example, the Fig. 4 shows a model of gene network composed by molecular triggers which realizes addition of binary numbers.

We assume here, that activity of the first genetic element of this trigger and inhibited state of the second one means “1”, and vice versa, “0” corresponds to activity of the second element, and inhibited state of the first one.

Figure 4b shows the Fragment 1 which calculates the sum of least lower-order digits of two binary numbers. Figure 4c corresponds to Fragment 2 which realizes summation of higher-order digits. In order to construct a gene network which adds two *n*-digit binary numbers, one has to join one Fragment 1 and (*n*−1) Fragments 2, as it is shown on the Fig. 4d for *n*=8. We assume here that different genetic elements of this network encode different proteins, so each regulatory binding is realised only by the proteins which correspond to the arrows of this Figure.

As an illustration, consider the logical sum 10010010+11001001=101011011, see Fig. 4e. Here, the triggers {*T*_1_} should be arranged according to the digits of the first summand, and the triggers {*T*_2_}, respectively, to the digits of the second one. This can be done by corresponding protein addition to the medium. Then, the regulatory binding bring this gene network into a state where the triggers {*S*} desribe the required sum. Here, activity of the last trigger in {*S*} corresponds to the highest-order digit of this sum.

Modeling of similar gene networks and corresponding experiments can be used in construction of artificial genetic systems, in particular, in design of specialized genetic computers.

4. Nonstationary dynamics in models of circular gene networks with negative feedback mechanisms appears for high level of the Hill’s parameter *h*. In the case of eukaryotes and prokaryotes, the necessary value of this nonlinearity parameter is achieved by multimerization of the transcriptional factors and/or by presence of several binding sites in DNA.

5. Nonstationary dynamics in these circuits is provided by the phenomenon of regulatory signal delay. In eukaryotes, this regulatory delay is achieved by cascade reactions in signaling pathways, separation of mRNA and protein synthesis into different compartments and transport of substances from one compartment to another. For one eukaryotic system, somitogenesis of vertebrata, as it is well-known, its development is regulated by an auto-oscillating process, controlled by autorepressor HES7 [24, 54–56]. Decreasing of the delay of autorepression signal of the gene *HES7* as a result of introns deletion for this gene, leads to distortions of oscillation period, and even to their disappearance, if all the introns are removed [57, 58].

Analysis of stability of oscillations in this system shows that the cooperativity coefficient *h* for HES1 self-repression should be sufficiently large (i.e. *h*≥4) and the duration of the repression loop is between 40 and 60 min [59].

In the case of the prokaryotes, there is a great deal of examples of the negative autoregulation of gene expression efficiency [1–9], but no examples of stable self-oscillating functioning regimes in these autorepressilators are known.

Thus, it is natural to suppose that for prokaryotes, due to the coupling of transcription and translation processes, the time period between the beginning of gene expresion and appearence of the active form of the transcriptional factor is too short for formation of oscillations.

Analysis of dynamics of synthetic constructions based on the prokaryotic genes and regulatory circuits with both, positive and negative loops shows that the regulatory signal delay that appears due to the negative autoregulatory loop is the key element of the oscillating construction design [60]. Moreover, numerical experiments show that extension of this loop leads to stable oscillations even in absence of positive regulatory loops [39].

From a theoretical viewpoint, the necessary value of the time lag can be realized by moving of the regulatory factor translation frame far enough from the transcription start. This can be done by long nucleotide sequence insertion between the promoter and transcription factor gene encoding. So, transcription of such long segments will imply the delay of appearence of the end product. Variations of the lengths of these insertions allow to control the time lag of the process.

This approach can be used in construction of synthetic gene networks with target functions. Corresponding mathematical models allow to find out structural, functional and parametric characteristics which provide necessary properties of the gene networks and give possibilities to plan strategy of their genetic synthesis.

## Conclusions

We have proposed two algorithms of construction and localization of limit cycles in these gene networks models. Existence and stability of some of these cycles have been shown analytically [45], and for this gene networks model we have solved an inverse problem of parameters identification. Several natural questions on behavior of trajectories of the system (12) remain still open. In particular, stability of cycles of this system is not proved; here, we have just some results of numerical experiments.

The modeling results demonstrate that non-stationary dynamics in the models of circular gene networks with negative feedback loops is achieved by a high degree of non-linearity of the mechanism of the autorepressor influence on its own expression, by the presence of regulatory signal delay, the value of which must exceed a certain critical value, and transcription/translation should be initiated from a sufficiently strong promoter/Shine-Dalgarno site. We believe that the identified patterns are key elements of the oscillating construction design.

Existence of asymmetric limit cycles in the symmetric systems of the types (3) and (12) is also unclear. We plan to consider these questions in our forthcoming studies.

### Methods

The main aim of our investigations is nonlocal analysis of some gene networks models represented by dynamical systems of kinetic type, and combimatorial structure of their phase portraits. In particular, we study here conditions of existence, stability, and (non)-uniqueness of cycles of the models as well as periods of these cycles, and their geometric location in phase portraits of the dynamical systems.

**Description of a model of simplest autorepressilator**

The equation

$$\frac{d x(t)}{dt} = f(x(t-\tau)) - x(t); \quad f(x) = \frac{\alpha}{1+x^{h}}; $$

corresponds to a subsystem containing just one gene that encodes protein with concentration *x*=*x*(*t*) acting as the regulator of its own expression by repression mechanism (autorepressor). The parameters of this model (1) of the simplest circular regulatory circuit are interpreted as follows: the constant *α* describes efficiency of the gene expression, *h* is a Hill coefficient, it determines degree of non-linearity of positive monotonically decreasing function *f*, which describes negative feedback mechanism of gene expression regulation. The parameter *τ* is the lag in the delay of the argument, the subtrahend in this equation and in higher-dimensional models corresponds to velocity of natural loss in the network (degradation, dissipation etc).

**Methods of analysis of higher-dimensional models**

Our analysis of phase portraits of the higher-dimensional gene networks models with negative feedback loops, delayed arguments phenomena, and studies of corresponding inverse problems were based on classical methods of the qualitative theory of ordinary differential equations, and on our recent results [44, 45] concerning monotonicity of the Poincaré map (the so-called succession map). Geometric arguments, topological fixed point theorem, and analytical calculations allow to prove stability of cycles and their non-uniqueness for some low-dimensional gene networks models.

At the same time, these geometric considerations show that phase portraits of *n*-dimensional dynamical systems of the type (10) contain invariant domains which can be decomposed to unions of 2^*n*^ parallelepipeds (blocks) such that solutions of such systems have very simple analytic form in each block (Fig. 2). So, the parameters identification inverse problems for the systems (10) can be reduced to combinatorial description of transitions of their trajectories from block to block. In the case *n*=3, the solution is given in Proposition 1. Higher-dimensional versions of this inverse problem require more combinatorial efforts even in the case *n*=4, see [49].

**Calculation method**

The methods of integrating the systems of differential equations with delayed arguments are classical as well, see [28, 30]. In our numerical experiments with these systems, we used the semi-implicit difference scheme with variable step. Corresponding calculations and construction of the graphs on the Figs. 2 and 3 which illustrate our mathematical results were carried out on personal computers of the first author.

## Data Availability

All data generated or analyzed during this study are included in this published article.

## Abbreviations

AMPK-mTOR-NRF2: AMPK (adenosine monophosphate-activated protein kinase)
mTOR: Mammalian target of rapamycin
NRF2: Nuclear factor erythroid 2 related factor 2
bHLH: Basic helix-loop-helix factor
cAMP: Cyclic adenosine monophosphate
ComK: Competence transcription factor
CRP: Cyclic receptor protein
Fis: Factor for inversion stimulation
FNR: Fumarate nitrate reduction regulator
H-NS: Histone-like nucleoid structuring protein
HES: Hairy and enhancer of split
IHF: Integration host factor
LexA: Locus for X-ray sensitivity A
Lrp: Leucine-responsive regulatory protein
MarR: Multiple antibiotic resistance regulator
RB: Retinoblastoma

## References

[1] Aiba H (1983). Autoregulation of the Escherichia coli crp gene: CRP is a transcriptional repressor for its own gene. Cell.

[2] Takahashi K, Hattori T, Nakanishi T, Nohno T, Fujita N, Ishihama A, Taniguchi S (1994). Repression of in vitro transcription of the Escherichia coli fnr and nar X genes by FNR protein. FEBS Lett.

[3] González-Gil G, Kahmann R, Muskhelishvili G (1998). Regulation of crp transcription by oscillation between distinct nucleoprotein complexes. EMBO J.

[4] Brent R, Ptashne M (1980). The lexA gene product represses its own promoter. Proc Natl Acad Sci USA.

[5] Prajapat MK, Jain K, Saini S (2015). Control of MarRAB operon in Escherichia coli via autoactivation and autorepression. Biophys J.

[6] Falconi M, Higgins NP, Spurio R, Pon CL, Gualerzi CO (1993). Expression of the gene en-coding the major bacterial nucleotide protein H-NS is subject to transcriptional auto-repression. Mol Microbiol.

[7] Aviv M, Giladi H, Schreiber G, Oppenheim AB, Glaser G (1994). Expression of the genes coding for the Escherichia coli integration host factor are controlled by growth phase, rpoS, ppGpp and by autoregulation. Mol Microbiol.

[8] Wang Q, Wu J, Friedberg D, Plakto J, Calvo JM (1994). Regulation of the Escherichia coli lrp gene. J Bacteriol.

[9] Pratt TS, Steiner T, Feldman LS, Walker KA, Osuna R (1997). Deletion analysis of the fis promoter region in Escherichia coli: antagonistic effects of integration host factor and Fis. J Bacteriol.

[10] Shan B, Chang CY, Jones D, Lee WH (1994). The transcription factor E2F-1 mediates the autoregulation of RB gene expression. Mol Cell Biol.

[11] Hirata H, Yoshiura S, Ohtsuka T, Bessho Y, Harada T, Yoshikawa K, Kageyama R (2002). Oscillatory expression of the bHLH factor Hes1 regulated by a negative feedback loop. Science.

[12] Bonev B, Stanley P, Papalopulu N (2012). MicroRNA-9 Modulates Hes1 ultradian oscillations by forming a double-negative feedback loop. Cell Rep.

[13] Navarro P, Festuccia N, Colby D, Gagliardi A, Mullin NP, Zhang W, Karwacki-Neisius V, Osorno R, Kelly D, Robertson M, Chambers I (2012). OCT4/SOX2-independent Nanog autorepression modulates heterogeneous Nanog gene expression in mouse ES cells. EMBO J.

[14] Fidalgo M, Faiola F, Pereira CF, Ding J, Saunders A, Gingold J, Schaniel C, Lemischka IR, Silva JC, Wang J (2012). Zfp281 mediates Nanog autorepression through recruit-ment of the NuRD complex and inhibits somatic cell reprogramming. Proc Natl Acad Sci USA.

[15] Trieu M, Ma A, Eng SR, Fedtsova N, Turner EE (2003). Direct autoregulation and gene dosage compensation by POU-domain transcription factor Brn3a. Development.

[16] Magenheim J, Hertz R, Berman I, Nousbeck J, Bar-Tana J (2005). Negative autoregulation of HNF-4alpha gene expression by HNF-4alpha1. Biochem J.

[17] Monteiro R, Pouget C, Patient R (2011). The gata1/pu.1 lineage fate paradigm varies between blood populations and is modulated by tif1 *γ*. EMBO J.

[18] Foka P, Singh NN, Salter RC, Ramji DP (2009). The tumour necrosis factor-alpha-mediated suppression of the CCAAT/enhancer binding protein-alpha gene transcription in hepato-cytes involves inhibition of autoregulation. Int J Biochem Cell Biol.

[19] Zhang H, Zhang Y, Chen C, Zhu X, Zhang C, Xia Y, Zhao Y, Andrisani OM, Kong L (2018). A double-negative feedback loop between DEAD-box protein DDX21 and Snail regulates epithelial-mesenchymal transition and metastasis in breast cancer. Cancer Lett.

[20] Gamba P, Jonker MJ, Hamoen LW (2015). A novel feedback loop that controls bimodal expression of genetic competence. PLoS Genet.

[21] Delihas N (2012). Regulating the regulator: MicF RNA controls expression of the global regulator Lrp. Mol Microbiol.

[22] Holmqvist E, Unoson C, Reimeg ard J, Wagner EG (2012). A mixed double negative feedback loop between the sRNA MicF and the global regulator Lrp. Mol Microbiol.

[23] Kapuy O, Papp D, Vellai T, Bánhegyi G, Korcsmáros T (2018). Systems-level feedbacks of NRF2 controlling autophagy upon oxidative stress response. Antioxidants (Basel).

[24] Kageyama R, Niwa Y, Isomura A (2012). González A, Harima Y. Oscillatory gene expression and somitogenesis. Wiley Interdiscip Rev Dev Biol.

[25] Tsai TY, Choi YS, Ma W, Pomerening JR, Tang C, Ferrell JR (2008). Robust, tunable biological oscillations from interlinked positive and negative feedback loops. Science.

[26] Shen S, Ma Y, Ren Y, Wei D (2016). Construction of an oscillator gene circuit by negative and positive feedbacks. J Microbiol Biotechnol.

[27] Edwards R, Glass L (2000). Combinatorial explosion in model gene networks. Chaos.

[28] Khlebodarova TM, Kogai VV, Fadeev SI, Likhoshvai VA (2017). Chaos and hyperchaos in simple gene network with negative feedback and time delays. J Bioinform Comput Biol.

[29] Suzuki Y, Lu M, Ben-Jacob E, Onuchic JN (2016). Periodic, quasi-periodic and chaotic dynamics in simple gene elements with time delays. Sci Rep.

[30] Kogai VV, Likhoshvai VA, Fadeev SI, Khlebodarova TM (2017). Multiple scenarios of transition to chaos in the alternative splicing model. Int J Bifurcat Chaos.

[31] Edwards R, Siegelmann HT, Aziza K, Glass L (2001). Symbolic dynamics and computation in model gene networks. Chaos.

[32] Likhoshvai VA, Kogai VV, Fadeev SI, Khlebodarova TM (2016). Chaos and hyperchaos in a model of ribosome autocatalytic synthesis. Sci Rep.

[33] Likhoshvai VA, Fadeev SI, Kogai VV, Khlebodarova TM (2013). On the chaos in gene networks. J Bioinform Comput Biol.

[34] Likhoshvai VA, Kogai VV, Fadeev SI, Khlebodarova TM (2015). Alternative splicing can lead to chaos. J Bioinform Comput Biol.

[35] Fadeev SI, Likhoshvai VA (2003). On hypothetical gene networks. J Appl Indust Math Sib Zh Ind Mat.

[36] Gardner TS, Cantor CR, Collins JJ (2000). Construction of a genetic toggle switch in Escherichia coli. Nature.

[37] Tchuraev RN, Stupak IV, Tropynina TS, Stupak EE (2000). Epigenes: design and construction of new hereditary units. FEBS Lett.

[38] Elowitz MB, Leibler S (2000). A synthetic oscillatory network of transcriptional regulators. Nature.

[39] Maeda K, Kurata H (2018). Long negative feedback loop enhances period tunability of biological oscillators. J Theor Biol.

[40] Sun M, Cheng X, Socolar JE (2013). Causal structure of oscillations in gene regulatory networks: Boolean analysis of ordinary differential equation attractors. Chaos.

[41] Glyzin SD, Kolesov AY, Rozov NK (2018). Quasi-Stable Structures in Circular Gene Networks. Comput Math Math Phys.

[42] Golubyatnikov VP, Minushkina LS (2019). Monotonicity of the Poincaré mapping in some models of circular gene networks. J Appl Ind Math.

[43] Golubyatnikov VP, Golubyatnikov IV, Likhoshvai VA (2010). On the Existence and stability of cycles in five-dimensional Models of Gene Networks. Numer Anal Appl.

[44] Golubyatnikov VP, Ivanov VV (2018). Cycles in odd-dimensional models of circular gene networks. J Appl Ind Mat.

[45] Golubyatnikov VP, Ivanov VV (2018). Uniqueness and stability of a cycle in 3-dimensional block-linear circular gene network models. Sib J Pure Appl Math.

[46] Akinshin AA, Golubyatnikov VP, Golubyatnikov IV (2013). On some multidimensional models of gene network functioning. J Appl Ind Mat.

[47] Glass L, Pasternack JS (1978). Stable oscillations in mathematcal models of biological control systems. J Math Biol.

[48] De Jong H, Gouze JL, Hernandez C, Page M, Sari T, Geiselmann J (2004). Qualitative simulation of genetic regulatory networks using piecewise-linear models. Bull Math Biol.

[49] Ayupova NB, Golubyatnikov VP (2015). On two classes of nonlinear dynamical systems: The four-dimensional case. Sib Mat J.

[50] Ayupova NB, Golubyatnikov VP (2014). On the uniqueness of a cycle in an asymmetric three-dimensional model of a melecular repressilator. J Appl Ind Mat.

[51] Tieri P, Farina L, Petti M, Astolfi L, Paci P, Castiglione F (2019). Network Inference and Reconstruction in Bioinformatics. Reference Module in Life Sciences. Encycl Bioinforma Comput Biol.

[52] Delgado FM, Gómez-Vela F (2019). Computational methods for Gene Regulatory Networks reconstruction and analysis: A review. Artif Intell Med.

[53] Akinshin AA, Golubyatnikov VP (2012). On cycles in symmetric dynamical systems. Bull Novosibirsk State Univ.

[54] Hirata H, Bessho Y, Kokubu H, Masamizu Y, Yamada S, Lewis J, Kageyama R (2004). Instability of Hes7 protein is crucial for the somite segmentation clock. Nat Genet.

[55] Mara A, Holley SA (2007). Oscillators and the emergence of tissue organization during zebrafish somitogenesis. Trend Cell Biol.

[56] Harima Y, Kageyama R (2013). Oscillatory links of Fgf signaling and Hes7 in the segmentation clock. Curr Opin Genet Dev.

[57] Takashima Y, Ohtsuka T, Gonzalez A, Miyachi H, Kageyama R (2011). Intronic delay is essential for oscillatory expression in the segmentation clock. Proc Natl Acad Sci USA.

[58] Harima Y, Takashima Y, Ueda Y, Ohtsuka T, Kageyama R (2012). Accelerating the tempo of the segmentation clock by reducing the number of introns in the Hes7 gene. Cell Rep.

[59] Bernard S, Cajavec B, Pujo-Menjouet L, Mackey MC, Herzel H (2006). Modelling transcriptional feedback loops: the role of Gro/TLE1 in Hes1 oscillations. Philos Trans A Math Phys Eng Sci.

[60] Stricker J, Cookson S, Bennett MR, Mather WH, Tsimring LS, Hasty J (2008). A fast, robust and tunable synthetic gene oscillator. Nature.

